# Reintroduction of immunosuppressive medications in pediatric rheumatology patients with histoplasmosis: a case series

**DOI:** 10.1186/s12969-021-00581-7

**Published:** 2021-06-07

**Authors:** Rachel A. Brown, Fatima Barbar-Smiley, Cagri Yildirim-Toruner, Monica I. Ardura, Stacy P. Ardoin, Shoghik Akoghlanian

**Affiliations:** 1 The Ohio State University College of Medicine, OH Columbus, USA; 2 Nationwide Children’s Hospital, Ohio Columbus, USA; 3 Department of Pediatric Rheumatology, Nationwide Children’s Hospital, 555 S. 18th St, OH 43205 Columbus, USA

**Keywords:** histoplasmosis, juvenile idiopathic arthritis, immunosuppression, tumor necrosis factor alpha inhibitor, corticosteroid, abatacept, DMARD, Cushing’s syndrome

## Abstract

**Background:**

Children with rheumatic diseases (cRD) receiving immunosuppressive medications (IM) are at a higher risk for acquiring potentially lethal pathogens, including *Histoplasma capsulatum* (histoplasmosis), a fungal infection that can lead to prolonged hospitalization, organ damage, and death. Withholding IM during serious infections is recommended yet poses risk of rheumatic disease flares. Conversely, reinitiating IM increases risk for infection recurrence. Tumor necrosis factor alpha inhibitor (TNFai) biologic therapy carries the highest risk for histoplasmosis infection after epidemiological exposure, so other IM are preferred during active histoplasmosis infection. There is limited guidance as to when and how IM can be reinitiated in cRD with histoplasmosis. This case series chronicles resumption of IM, including non-TNFai biologics, disease-modifying anti-rheumatic drugs (DMARDs), and corticosteroids, following histoplasmosis among cRD.

**Case presentation:**

We examine clinical characteristics and outcomes of 9 patients with disseminated or pulmonary histoplasmosis and underlying rheumatic disease [juvenile idiopathic arthritis (JIA), childhood-onset systemic lupus erythematosus (cSLE), and mixed connective tissue disease (MCTD)] after reintroduction of IM. All DMARDs and biologics were halted at histoplasmosis diagnosis, except hydroxychloroquine (HCQ), and patients began antifungals. Following IM discontinuation, all patients required systemic or intra-articular steroids during histoplasmosis treatment, with 4/9 showing Cushingoid features. Four patients began new IM regimens [2 abatacept (ABA), 1 HCQ, and 1 methotrexate (MTX)] while still positive for histoplasmosis, with 3/4 (ABA, MTX, HCQ) later clearing their histoplasmosis and 1 (ABA) showing decreasing antigenemia. Collectively, 8/9 patients initiated or continued DMARDs and/or non-TNFai biologic use (5 ABA, 1 tocilizumab, 1 ustekinumab, 3 MTX, 4 HCQ, 1 leflunomide). No fatalities, exacerbations, or recurrences of histoplasmosis occurred during follow-up (median 33 months).

**Conclusions:**

In our cohort of cRD, histoplasmosis course following reintroduction of non-TNFai IM was favorable, but additional studies are needed to evaluate optimal IM management during acute histoplasmosis and recovery. In this case series, non-TNFai biologic, DMARD, and steroid treatments did not appear to cause histoplasmosis recurrence. Adverse events from corticosteroid use were common. Further research is needed to implement guidelines for optimal use of non-TNFai (like ABA), DMARDs, and corticosteroids in cRD following histoplasmosis presentation.

## Background

Over 300,000 children in the USA live with juvenile idiopathic arthritis (JIA) and other chronic rheumatic diseases. Advances in understanding the cytokine-driven pathogenesis of rheumatic diseases have provided potent therapeutics. A growing number of FDA approved biologics targeting cytokines are available, including inhibitors of the pro-inflammatory mediators tumor necrosis factor alpha (TNFai), interleukin (IL)-1b (canakinumab), IL-1R (anakinra), IL-6R (tocilizumab), as well as T-cell co-stimulation (abatacept [ABA]) and B-cells (rituximab) [1]. However, cytokines are also critical to control and clear infections, and autoimmunity itself increases infection risk due to immune system dysregulation [1]. Immunosuppressive medications (IM) heighten risk of developing serious infections, including granulomatous infections like histoplasmosis [1–3]. Histoplasmosis is caused by inhaling spores of *Histoplasma capsulatum*, a fungus found in soil contaminated with bird or bat droppings and endemic to areas such as the Ohio and Mississippi River Valleys [4–6]. While typically causing mild or asymptomatic pulmonary infection in immunocompetent patients, *H. capsulatum* can disseminate systemically in susceptible hosts, leading to poor outcomes and requiring a prolonged antifungal course [6–8]. Furthermore, itraconazole, the recommended oral antifungal for *H. capsulatum*, can result in drug interactions due to inhibiting the CYP3A4 pathway [9–13].

Our center at Nationwide Children’s Hospital (NCH) in Columbus, Ohio facilitates over 6,000 visits per year for children with rheumatic diseases (cRD). Standard rheumatic treatments include nonsteroidal anti-inflammatory drugs (NSAIDs), corticosteroids, and disease-modifying anti-rheumatic drugs (DMARDs) (methotrexate [MTX], leflunomide, hydroxychloroquine [HCQ], etc.), but many of these patients are or were also managed with TNFa antagonists (etanercept, infliximab, adalimumab, etc.) or other biologics at some point. It is controversial whether low dose MTX increases infection susceptibility, whereas combination IM therapy, corticosteroids, or biologics do, while HCQ does not [1, 3, 14, 16, 17]. Indeed, TNFai complicated by infections is well reported [3, 8, 17–22]. For patients receiving TNFai who present with fever or pulmonary symptoms, extensive infectious workup is undertaken given the broad infectious differential. It is recommended that IM be held during acute infections, but this interruption in treatment may lead to a rheumatic disease flare. Optimal rheumatic disease management is unknown, including the duration of IM discontinuation and, importantly, safety data about reintroducing TNFai or other biologics are largely lacking [1, 3, 23, 24, 22]. Meta-analyses suggest some biologics carry a lower infection risk compared to TNFai [25]. For example, an observational study of adult patients with rheumatoid arthritis (RA) noted participants receiving ABA had lower rates of infection-related hospitalization compared with other biologics [26]. However, utilization of these non-TNFai following histoplasmosis diagnosis (FHD) and histoplasmosis clearance (HC) has not been reported and merits further scrutiny. In our report, we describe the treatment course, IM management, and outcomes of cRD diagnosed with histoplasmosis.

## Methods

A retrospective chart review of electronic health records identified children followed for rheumatic disease management at NCH, who were diagnosed with and treated for proven, probable, or possible histoplasmosis between January 2011 and January 2018. Primary inclusion criteria included: diagnosis with a pediatric rheumatic disease and a later/concurrent diagnosis of histoplasmosis. Data were collected for IMs prior to and following histoplasmosis diagnosis and histoplasmosis course. IMs were characterized as (1) biologic DMARDs, such as TNFai or non-TNFai, (2) non-biologic DMARDs, and (3) corticosteroids, including intra-articular steroid injections (IASIs) and oral/inhaled/intermuscular/intravenous (systemic) steroids. Systemic steroids were classified as “burst” (high dose > 10 mg/day provided to treat active rheumatic or non-rheumatic disease, like asthma) or “maintenance” (low dose ≤ 10 mg/day). Histoplasmosis resolution was defined as clearance of urine and serum antigens and clinical resolution of signs and symptoms. This study was reviewed and approved by the local Institutional Review Board IRB17-01241.

## Results

Nine cRD patients (78 % female) met inclusion criteria, including 7 (78 %) patients with JIA (2 psoriatic, 2 polyarticular, 2 enthesitis-related arthritis [ERA], and 1 oligoarticular), 1 childhood-onset systemic lupus erythematosus (cSLE), and 1 mixed connective tissue disease (MCTD) patient. Patient demographics and pre-histoplasmosis diagnosis medications are shown in Table 1. Five out of 7 JIA patients were receiving IM prior to infection diagnosis: 1 was receiving MTX alone and 4 were receiving combination therapy with MTX and TNFai (2 adalimumab, 1 etanercept, 1 infliximab), including one patient also receiving burst steroids. The other 2 patients with JIA were managed with NSAIDs. Both cSLE and MCTD patients were on MTX, HCQ, and systemic steroids (burst and maintenance, respectively). Median time from starting TNFai to histoplasmosis manifestation was 1.3 months (range 0.5–14.1) compared with 18 months (4.6–32) and 13 months (0.5–31) for DMARDs and steroids, respectively.

**Table 1:** Characteristics of patients at time of histoplasmosis diagnosis. Information was collected on patient demographics and IM prior to histoplasmosis diagnosis

Patient	Age (yr) at Histoplasmosis Diagnosis, Sex	Rheumatic Disease	Anti-TNFa (duration of Therapy in Months Prior to Histoplasmosis Diagnosis)	Other Pre-histoplasmosis IM (month duration)
1	11, F	JIA, Psoriatic	**Adalimumab (0.5)**	MTX (5)
2	17, F	JIA, Poly	**Etanercept (1.3)**	MTX (7), burst steroids (1)
3	18, F	JIA, Psoriatic	**Infliximab (14.1)**	MTX (23)
4	15, F	cSLE	-	MTX (3), HCQ (5), burst steroids (12.8)
5	16, F	JIA, Enthesitis	-	None
6	9, M	JIA, Enthesitis	**Adalimumab (1.3)**	MTX (15)
7	17, F	JIA, Oligo	-	MTX (18)
8	16, M	MCTD	-	MTX (31), HCQ (32), maintenance steroids (30)
9	5, F	JIA, Poly	-	None
Summary, % or Median (range):	16 [5–18], 78 % F	7 JIA, 1 cSLE, 1 MCTD	44 % TNFai, 1.3 (0.5–14.1)	78 % DMARD, 18 (4.6–32) 33 % Steroids, 13 (0.6–30)

Key: Pt = patient identifier; yr = years; IM = immunosuppressive medication; JIA = juvenile idiopathic arthritis, MTX = methotrexate, Poly = polyarticular; cSLE = childhood-onset systemic lupus erythematosus; HCQ = hydroxychloroquine; Oligo = oligoarticular; MCTD = mixed connective tissue disease

Histoplasmosis diagnosis and course are shown in Table 2. Three (33 %) patients were able to identify potential exposures (recent bonfire, farm residence, and birds/bats in attic). *H. capsulatum* was detected by bronchoalveolar lavage, pulmonary wedge biopsy, *Histoplasma* antigen, and/or serology for a total of 3 proven, 5 probable, and 1 possible histoplasmosis cases [27]. Five (56 %) patients developed disseminated histoplasmosis (DH), and 3 (33 %) developed pulmonary histoplasmosis (PH). The possible case was not further categorized as PH or DH but included in this series due to positive serology and clinical symptoms (data not shown). For the 5 DH patients, 2 were receiving both TNFai and MTX, 1 was receiving TNFai, MTX, and steroid bursts, 1 was receiving DMARDs only, and 1 was receiving DMARD and maintenance steroids. Of the 3 PH patients, 1 was receiving TNFai and MTX and 2 were not receiving IMs. The patient with the possible histoplasmosis infection was receiving DMARDs and burst steroids. At histoplasmosis diagnosis, IM, except HCQ and steroids, were held. Six (67 %) patients (5 DH and 1 PH) required hospitalization for acute histoplasmosis for a median of 14 days [6–22], with 1 DH patient needing admission to the intensive care unit for mechanical ventilation for 14 days. During follow up, 7/9 (78 %) demonstrated complete resolution of antigenemia and clinical symptoms with a median time to clearance of 12.7 months (2.3–37.2) FHD and 2 were clinically asymptomatic at 11 and 25 months FHD while showing low antigenemia. Four of nine (44 %) patients continued prophylactic itraconazole after HC for a median of 23.6 months (5.0–70.0). Median follow-up after HC was 29.3 months (0-70.3), with a total follow-up from histoplasmosis diagnosis of 33 months (11-88.5). There was no observed mortality or histoplasmosis relapse during this time.

**Table 2:** Histoplasmosis presentation. Data were collected for histoplasmosis infection, type, hospitalization, diagnostic tests, treatment, and outcomes

Pt	Known Exposure	Histoplasmosis Type	Histology Staining	Antigen (Serum, Urine)	Category	Days Hospitalized (Intensive care)	Antifungal Therapy	Outcome (months to HC or still antigen +) Itraconazole prophylaxis (months)	Months Follow Up After Histoplasmosis Diagnosis, Clearance
1 JIA	No	PH	ND	+,+	Probable	18	Amphotericin B Itraconazole	HC at 36.7 Prophylaxis for 19.6	80, 43.3
2 JIA	No	DH	+ BAL	+,+	Proven	11	Amphotericin B Itraconazole	HC at 37.2 Prophylaxis for 5.0	89, 51.8
3 JIA	No	DH	+ pulmonary wedge biopsy	+,+	Proven	22, (14, ventilation)	Amphotericin B Itraconazole	HC at 12.7 Prophylaxis for 70.0	83, 70.3
4 cSLE	No	Uncategorized	ND	-,-	Possible	No	Itraconazole*	HC at 3.7	33, 29.3
5 JIA	No	PH	ND	-,-	Probable	No	Itraconazole Fluconazole	HC at 2.3	28, 25.7
6 JIA	Lives on farm	DH	ND	+,+	Probable	17	Amphotericin B Itraconazole	HC at 21.2 Prophylaxis for 27.5	67, 45.8
7 JIA	Attended Bonfire	DH	ND	+,+	Probable	6	Itraconazole	Asymptomatic Antigen < LOQ at 18.0+ Halted itraconazole 18.0 FHD	26, NA
8 MCTD	No	DH	ND	+,+	Probable	8 (1**, No ventilation)	Amphotericin B Itraconazole	Asymptomatic Antigen decreasing at 11.0	11, NA
9 JIA	Birds and Bats in attic	PH	+ pulmonary wedge biopsy	-,-	Proven	No	Itraconazole	HC at 3.3	18, 14.7
Summary: Median (range)	33 % Yes 66 % No	56 % DH 33 % PD 11 % unnoted	3 Positive 6 ND	67 % Antigen + (100 % DH, 33 % PH)	3 Proven 5 Probable 1 Possible	67 % for 14 (6–22) 11 % needing ventilation	*89 % Itraconazole 56 % Amphotericin B 11 % Fluconazole	78 % now HC 12.7 to HC (2.3–37.2) 44 % prophylaxis for 23.6 (5–70)	FHD 33 (11-88.5) HC 29.3 (0-70.3)

*Given itraconazole, but declined to take it.**Intensive care stay during hospitalization was attributed to “possible shock 2nd to adrenal insufficiency.”

Key: Pt = Patient identifier and rheumatic disease; PH = Pulmonary histoplasmosis; DH = Disseminated histoplasmosis; ND = No data; + = Positive; - = Negative; HC = Histoplasmosis clearance; BAL = Bronchoalveolar lavage; LOQ = Limit of quantification; FHD = Following histoplasmosis diagnosis

### Patient Use of Immunosuppressive Medications Following Histoplasmosis Infection was Necessary

Patients initially managed their underlying rheumatic conditions during antifungal therapy with NSAIDs or HCQ, but while receiving these medications all patients experienced rheumatic disease flare requiring further intervention.

### Steroids

Prior to HC and while still taking itraconazole, all patients received steroidal therapy (Table 3), with 5/9 (56 %) receiving systemic steroids (including for non-rheumatic conditions or maintenance doses) and 8/9 (89 %) patients (7/7 JIA and 1 MCTD) receiving IASIs. Collectively, 4 received IASI and systemic steroids (including 1 continuing maintenance dosing), 4 received solely IASI, and 1 continued prior systemic steroid burst usage. Adverse events were common. One patient reported known corticosteroid allergies and developed sweating and chills limited to 24 h. Another 4 patients developed days to months of typical Cushingoid features shortly after IASIs, including both (2/2) patients receiving burst steroids and IASI as well as half (2/4) of the patients just receiving IASI without any systemic steroids. The median time FHD was 4.3 months (0–15.0) to starting systemic steroids (includes maintenance dosing) and 8.3 months (0.8–18.3) for IASI (Table 3).

**Table 3:** Use of intra-articular steroid injections (IASI) and systemic steroids and adverse events during histoplasmosis treatment. Data for number and timing of IASI and systemic steroids were collected as well as concomitant use of itraconazole. Adverse events were noted

Pt	IASI (months after histoplasmosis diagnosis)	Systemic Steroids (months after histoplasmosis diagnosis)	Concomitant Itraconazole?	Description of Adverse Event
1 JIA	3 (7.2)	Intermittent high/burst (5.0 on)	Yes	Red, puffy face (duration unclear)
2 JIA	18 (2.4)	Intermittent high/burst (4.3 on)	Yes	9 + months of weight gain, cushingoid facies, buffalo hump, protruding abdomen with striae, diabetes, adrenal insufficiency, and probable spinal compression fractures.
3 JIA	2 (12.4) and 3 (17.9)	No	Yes	None
4 cSLE	No	Bursts (0, never halted)	Yes	None
5 JIA	1 (1.1)	No	Yes	None
6 JIA	1 (18.3)	Systemic (15.0)	Yes	< 1 day of sweating, chills, and flushing (allergic reaction*)
7 JIA	2 (9.2)	No	Yes	2.5 + months of cushingoid facies
8 MCTD	3 (7.4)	Maintenance (0, never halted)	Yes	None
9 JIA	7 (0.8)	No	Yes	Around 9 months of weight gain, increased appetite, moon facies, and protruding abdomen
Summary:	89 % received IASI 8.3 (0.8–18.3)	56 % received systemic steroids 4.3 (0–15.0)	Yes	44 % experienced non-allergic adverse reactions to steroids*

*P6 has known steroid allergy

Key: Pt = Patient identifier and rheumatic disease, IASI = Intra-articular steroid injection

### Biologics and DMARDs

Active rheumatic disease led to initiating biologics and DMARDs in 7 patients (Table 4) including: 3 ABA (with 1 also continuing their prior HCQ); 1 ABA, MTX, and ustekinumab; 1 ABA and HCQ; 1 MTX and leflunomide; and 1 HCQ, MTX, and tocilizumab. One patient did not begin biologics nor DMARDs and another continued taking their prior HCQ. Collectively, six patients began the non-TNFai ABA (5) or tocilizumab (1) a median of 17.4 months (4.8–25.5) FHD, with 4 (3 ABA; 1 tocilizumab) patients having already resolved their histoplasmosis. One patient who was receiving ABA was transitioned to ustekinumab 33.6 months following HC (46.3 months FHD). Among the 2 patients still showing low, asymptomatic antigenemia at ABA initiation, 1 cleared their antigenemia after 37 months (16.6 after beginning ABA) and the other patient remained antigen positive at ≥ 18 months FHD, although still asymptomatic at 25.4 months FHD. Additionally, 6/9 (67 %) patients initiated DMARDs. MTX was started by 3 patients a median of 22 months (11.2–51.2) FHD, with 1 patient still asymptomatically antigen-positive (11 months FHD) and 2 patients already cleared (median 20 months FHD). This positive patient cleared their antigenemia 26 months after beginning MTX. HCQ was initiated in 2 antigen-positive, asymptomatic patients at 6 and 10 months FHD and prior HCQ was continued in 2 others. These 2 positive patients had resolution of their antigenemia 15 and 27 months after beginning HCQ, respectively. Leflunomide was started in one patient taking MTX 25 months after HC (62 months FHD). The median time FDH to first taking DMARDs was 3.1 months (0 [still positive]-51.2) and to first beginning biologics was 17.4 months (4.8–25.5), while the median time following HC to DMARD and biologic initiation was 0 (0 to 9.4) and 2 months (0–11), respectively (Table 4).

**Table 4:** Reintroduction of immunosuppressive medications and patient histoplasmosis course. Patient history was examined for timing of systemic immunosuppressive medication reinitiation (localized steroid use was not included). Timing of reinitiation was compared with state of histoplasmosis, with “still +” denoting patient had not cleared their histoplasmosis infection based on antigenemia and/or clinical symptoms and “HC for #” indicating how many months it had been since histoplasmosis clearance to beginning the immunosuppressive medication. Median months of follow-up since histoplasmosis clearance was 45.6 (14.7–78.9)

Pt	Immunosuppressive Medication Resumption (months following histoplasmosis diagnosis, histoplasmosis status) Months to HC			
1 JIA	HCQ (10.0, still +), ABA (20.1, still +), Intermittent systemic steroid bursts (5.0 on, still +) Time to HC: 36.7			
2 JIA	MTX (11.2, still +), Leflunomide (61.8, HC for 24.6), Stress/Intermittent steroid bursts (4.3, still +) Time to HC: 37.2			
3 JIA	MTX (22.1, HC for 9.4), ABA (14.2, HC for 1.4), Ustekinumab (46.3, HC for 33.6) Time to HC: 12.7			
4 cSLE	HCQ (0, still +), ABA (14.7, HC for 11.0), Low dose maintenance steroids (0, still +) Time to HC: 3.7			
5 JIA	ABA (4.8, HC for 2.5) Time to HC: 2.3			
6 JIA	HCQ (6.2, still +), MTX (51.2, HC for 30.0), Tocilizumab (21.8, HC for 4.9), Systemic steroids (15.0, HC for 0.6) Time to HC: 21.2			
7 JIA	ABA (25.5, still +) Antigen positive < LOQ at 18+			
8 MCTD	HCQ (0, still +), Low dose stress steroids (0, still +) Antigen positive and decreasing at 11+			
9 JIA	None Time to HC: 3.3			
Summary:	Total patients resuming IM: 8/9 (89), with 6/9 (67 %) DMARD; 6/9 (67 %) biologics; and 5/9 (56 %) systemic steroids	Collective median (range) after diagnosis: DMARDs 3.1 (0-51.2) Biologic 17.4 (4.8–25.5) Corticosteroid 4.3 (0–15.0)	Collective median (range) to IM use from HC: DMARD Still + (Still + to 9.4) Biologic 2 (Still + to 11.0) Corticosteroid all still +	Pt median (range) to HC: 12.7 (2.3–37.2), with 2 still low positives

Key: Pt = Patient identifier and rheumatic disease; IM = Immunosuppressive medication; HCQ = hydroxychloroquine; ABA = abatacept; MTX = methotrexate, HC = histoplasmosis clearance; LOQ = Limit of quantification; + = positive

## Discussion

In this case series, we describe treatment and disease course of cRD diagnosed with histoplasmosis. Outcomes were favorable, with non-TNFai biologic and DMARD medications, including ABA and HCQ, being safely reinitiated in patients requiring rheumatic disease alleviation. Although TNFai therapy complicated by granulomatous infections like histoplasmosis has been well-recognized [7, 17–20, 23], few published reports address reinstitution of IM treatments following histoplasmosis diagnosis [8, 19, 22, 23]. Moreover, there is a lack of guidance on underlying disease management and long-term follow-up [19, 22]. In this series, rheumatic diseases were initially controlled by NSAIDs and HCQ, but all cRD eventually needed additional treatments. Moreover, we observed that Cushingoid symptoms developed in almost half (4/9) of patients receiving corticosteroids during antifungal treatment. Thus, managing rheumatic disease during recovery from histoplasmosis poses challenges as IM are halted upon serious infections.

### Itraconazole and Steroids

The often-chronic nature of fungal and rheumatic therapies make selecting compatible medications critical. In severe DH, initial therapy may begin with intravenous amphotericin transitioning to oral itraconazole, with a total antifungal duration of at least 12 months [7, 21]. Itraconazole is an effective antifungal, but it potently inhibits the CYP3A4 pathway, thereby decreasing steroid metabolism [8, 9, 11, 12]. Nevertheless, adjunctive corticosteroids may still be indicated for fungal infections and other inflammatory states, like rheumatic flares [1, 7, 8]. Furthermore, systemic or local corticosteroid use by itself can also cause iatrogenic Cushing’s syndrome, although drug interactions increase risk [9, 28, 29]. Shortly following IASIs, 44 % of our cRD developed non-allergic adverse reactions with prominent Cushingoid features. To our knowledge, this is the first description of Cushing’s syndrome in cRD undergoing itraconazole treatment following IASI and systemic steroids. However, previous reports associating itraconazole and steroid therapy with hypothalamic-pituitary-adrenal (HPA) axis suppression and, more infrequently, overt Cushing’s syndrome have been published, usually for patients with cystic fibrosis taking inhaled steroids [9, 10, 13, 15].

The somewhat surprisingly high fraction of patients presenting with overt Cushingoid features in our report could perhaps be due to differences in systemic steroid levels, route of steroid administration, type of steroid, underlying disease, and/or drug interactions. For example, a study comparing the effects of itraconazole on a single dose of methylprednisolone versus prednisone in healthy volunteers indicated prednisone’s pharmacokinetics were unaffected while methylprednisolone concentration increased [12]. In our limited cohort, is difficult to draw firm conclusions about what/if combinations of corticosteroids and itraconazole place patients at greatest risk for HPA axis-related syndromes. High dose steroidal bursts combined with IASI perhaps contribute, but more research is needed, for example, to determine if route of steroid administration matters and if underlying comorbidities affect risk. Regardless, there is an urgency to determine optimal strategies of IM usage in the setting of concurrent histoplasmosis that facilitate histoplasmosis recovery and rheumatic disease management. Moreover, perhaps HPA axis monitoring should be considered in patients needing itraconazole and steroids [13].

### Histoplasmosis and Immunosuppressants

Acute DH develops in about 1 of every 2000 acute histoplasmosis cases, and most acute DH occurs in immunosuppressed patients [30]. Markedly, immunocompromised patients (e.g., AIDS) have a 10-fold increased risk for developing DH compared to immunocompetent persons, and DH mortality rates in severely immunocompromised patients can reach 30 % [31–33]. Chu et al. noted that in 111 pediatric cases of histoplasmosis requiring hospitalization, 32 % of these patients were immunocompromised and/or had a comorbidity with an overall pediatric mortality of 5 % [33]. Likewise, a review of 73 pediatric histoplasmosis cases by Ouellette et al. reported immunocompromised children had a 2.5X higher rate (56 % vs. 21 %) of developing DH [34]. In line with these findings, in our series most (66 %) PH cases were in patients without prior IM, while all patients with DH were receiving prior combination or DMARD therapy.

Studies assessing post-histoplasmosis care in patients experiencing TNFai complicated by histoplasmosis are limited [19, 20, 22, 23]. Notably, Vergidis et al. described outcomes of 98 patients with various autoimmune diseases diagnosed with histoplasmosis, wherein 3/98 patients continued non-combination TNFai, 11/58 patients continued DMARDs, and 13/23 patients continued corticosteroids. The 3 histoplasmosis cases maintaining TNFai, which is not the standard of care, were described as “mild” and all patients achieved remission after 6–10 months of itraconazole. During follow-up (median 32 months), 2/25 patients resuming TNFai (and 1/49 who had not) developed histoplasmosis recurrence with 1 fatality (taking TNFai). Follow-up data were unavailable for the other patients, and resumption information on DMARDs and corticosteroids was not reported [22]. Furthermore, after TNFai was held in 14/15 adult patients with RA, 4 resumed them at least 5 months FHD. The patient continuing TNFai, whose lung biopsy showed inactive disease, did well, while one patient who resumed TNFai developed clinical recurrence, permanently halted it, and recovered [23]. In our case series, persistent rheumatic disease activity led to resumption of biologics and non-HCQ DMARDs in 1 cSLE and 6 JIA patients FHD. However, no patients were restarted on TNFai due to safety concerns even if histoplasmosis disease was mild. One JIA patient did not require IM and the patient with MCTD proceeded on their pre-histoplasmosis regimen of HCQ and maintenance steroids. The 3 patients resuming MTX had favorable outcomes. HCQ was continued without issue for the cSLE and MCTD patients, and HCQ was an alternative (or additive) treatment option for JIA patients. Unlike some evidence on MTX, HCQ does not increase infection risk, but all 3 patients starting either medicine cleared their histoplasmosis without recurrence.

Considering timing of biologic reinitiation FHD, 5 cRD began the non-TNFai ABA before [2] or following [3] HC, while the initiations of tocilizumab or ustekinumab were only after HC. Although no patients were receiving ABA, tocilizumab, or ustekinumab prior to their histoplasmosis diagnosis, all 4 cRD beginning them have remained free of histoplasmosis recurrence and neither patient starting ABA while still antigen positive experienced worsening of their histoplasmosis. Moreover, 1 of these 2 ABA patients has achieved HC to date. Despite the small numbers, the use of ABA in these cRD resulted in good rheumatologic outcomes without exacerbating the infection, suggesting that ABA may be an option for treating rheumatic patients with histoplasmosis. The inability to support some cRD without higher tier medications underscores the need to promote infection safety while controlling rheumatic disease. For example, considering lower risk (non-TNFai) biologics, like ABA, for patients in endemic fungal areas such as the Ohio and Mississippi River Valleys where histoplasmosis is the most frequent TNFai-associated endemic fungal infection [6]. While screening for histoplasmosis prior to beginning biologics is not routinely recommended, nevertheless physicians prescribing IM must be aware of histoplasmosis presentations and risk factors [1].

## Conclusions

Histoplasmosis is an important complication of the immunosuppressive therapy utilized to treat cRD in endemic areas. This small case series proposes corticosteroids, non-HCQ DMARDs, and non-TNFai biologic treatments initiated months FHD or after HC did not appear to induce histoplasmosis recurrence, and, for a majority of patients, permitted histoplasmosis antigen clearance. While receiving antifungal therapy, necessary intra-articular or systemic steroid usage may potentially merit HPA axis monitoring. However, firm conclusions from our report are limited due to the small number of patients and varying IM therapies. Given the crucial role of IMs like biologics and DMARDs in cRD care, additional studies are needed to examine the long-term safety and efficacy of ABA, tocilizumab, ustekinumab, MTX, HCQ, and corticosteroids during histoplasmosis treatment and recovery.

## Data Availability

The datasets analyzed during the current study available from the corresponding author on reasonable request.

## Abbreviations

JIA: Juvenile idiopathic arthritis
TNFai: Tumor necrosis factor alpha inhibitor
IL-1b: Interleukin-1 beta
IL-1R: Interleukin-1 receptor
IL-6R: Interleukin-6 receptor
ABA: Abatacept
IM: Immunosuppressive medications
*H*.* capsulatum*: Histoplasmosis capsulatum
cRD: Children with rheumatic disease
NSAIDs: Nonsteroidal anti-inflammatory drugs
DMARDs: Disease-modifying anti-rheumatic drugs
MTX: Methotrexate
HCQ: Hydroxychloroquine
RA: Rheumatoid arthritis
FHD: Following histoplasmosis diagnosis
HC: Histoplasmosis clearance
NCH: Nationwide Children’s Hospital
IASIs: Intra-articular steroid injections
ERA: Enthesitis-related arthritis
cSLE: Childhood-onset systemic lupus erythematosus
MCTD: Mixed connective tissue disease
DH: Disseminated histoplasmosis
PH: Pulmonary histoplasmosis
HPA: Hypothalamic-pituitary-adrenal
